# The detection of urinary viruses is associated with aggravated symptoms and altered bacteriome in female with overactive bladder

**DOI:** 10.3389/fmicb.2022.984234

**Published:** 2022-09-23

**Authors:** Qi Sun, Leqian Li, Hao Zhou, Ying Wu, Yubo Gao, Bingyi Wu, Yifeng Qiu, Zhipeng Zhou, Qixiang Song, Jie Zhao, Peng Wu

**Affiliations:** ^1^Department of Urology, Nanfang Hospital, Southern Medical University, Guangzhou, China; ^2^Department of Hospital Infection Management, Nanfang Hospital, Southern Medical University, Guangzhou, China; ^3^School of Public Health, Southern Medical University, Guangzhou, China; ^4^Medical Research Center, Nanfang Hospital, Southern Medical University, Guangzhou, China; ^5^Department of Urology, Renji Hospital, Shanghai Jiao Tong University School of Medicine, Shanghai, China; ^6^School of Pharmaceutical Sciences, Southern Medical University, Guangzhou, China

**Keywords:** female urinary microbiome, overactive bladder, metagenome, virome, microbiota

## Abstract

Although it is known that changes in bacterial components of the urinary microbiome are associated with overactive bladder (OAB), the specific role of viruses is still insufficiently investigated. The aim of the present study is to evaluate the role of urinary viruses in woman with OAB, and analyze the potential relationship between viruses, bacteria and disease. Catheterized urine samples were collected from 55 women with OAB and 18 control individuals. OAB patients fulfilling the following criteria were considered eligible for this study: female, 18 years of age or older; presented with classic OAB symptoms defined by the International Continence Society; and OAB Symptom Score (OABSS) total score ≥ 3 points and question 3 (urgency) score ≥ 2 points. Based on results of metagenomic next-generation sequencing (mNGS), all participants were divided into virus-infected and virus-uninfected groups for analysis. The results of mNGS showed that the diversity of the OAB group was lower than that of the control group when focused on bacterial sequences, which was consistent with our previous study. According to the questionnaire filled out by the patients, OABSS and 8-item OAB questionnaire, female OAB patients who had viruses detected in their urine had more severe symptoms. In parallel, John Cunningham virus (mainly subtype 7 and subtype 2) was the most frequently detected virus in urine. Correlation analysis indicated that risk factors for virus infection in OAB patients include age, habit of holding urine and pelvic surgery history. Given our preliminary data, viral infection can aggravate OAB severity and affect the composition of bacterial. Further research is required to explain how viral infections can aggravate OAB patient symptoms and cause bacterial changes.

## Introduction

Overactive bladder (OAB) is characterized as “urgency, with or without urgency urinary incontinence (UUI), usually with increased daytime frequency and nocturia” defined by the International Continence Society (ICS; Abrams et al., 2003). The pioneering works of Brading suggested that abnormal electrical couping of smooth muscle cells resulting in detrusor contraction while other researches suggesting sensation of urgency may be provoked by urothelial/suburothelial dysfunction (Drake et al., 2001; Lee et al., 2010; Chen et al., 2017). It has been proposed that metabolic syndrome, affective disorders, sex hormone deficiency, urinary microbiota, gastrointestinal dysfunction and subclinical autonomic nervous system dysfunction are potential pathophysiological cofactors of OAB (Peyronnet et al., 2019). However, there are still a number of patients failed to respond to the medications, including antimuscarinics and the β3-adrenoreceptor agonist, requiring invasive interventions (Wadie, 2014; De Wachter et al., 2019). Therefore, the multifactorial nature of OAB and the effective pharmacotherapy of refractory cases compose the two main obstacles in basic science research and clinical management.

Emerging evidence indicated that alteration to urinary microbiome is closely related to the pathogenesis of OAB using 16S rRNA gene sequencing and expanded quantitative urine culture (EQUC) (Price et al., 2016; Gasiorek et al., 2019; Lundy et al., 2021). Wolfe et al. identified urinary bacteria community with 16S rRNA sequencing in female patients undergoing urogynecologic surgery with negative standard urine cultures, and developed its linkage with diverse urinary diseases (Wolfe et al., 2012). Pearce et al. (2014) used EQUC and 16S rRNA sequencing to compare the urinary microbiome of women with and without wet OAB (UUI) and their data suggest a potentially important difference in the urinary microbiome of women in the two groups. Combined, with the in-depth research on the urinary microbiome, from sterile to fertile, a growing number of scholars realize that the current research results are just the tip of the iceberg (Hilt et al., 2014; Price et al., 2016; Brubaker et al., 2021).

The urinary microbiome, however, contains not just bacteria, but also viruses, fungi, parasites, and other species (Lloyd-Price et al., 2016). Incidentally, excluding the bacteria, studies on other microorganisms, particularly viruses and their association with OAB are limited (Brubaker et al., 2021). Among them, most frequently detected viruses in urinary tract are human polyomavirus type 1 (BK Virus) and 2 (JC Virus), both of which are mainly studied in immunocompromised patients, such as kidney and bone marrow transplant recipients (Jhang et al., 2018). Meanwhile, both JC and BK are double-stranded DNA viruses and belong to polyomavirus (Salabura et al., 2021). Detection of JC Virus is reported in recent studies of urinary diseases such as lower urinary tract symptoms (LUTS) and OAB, but its relationships with urinary bacteria and OAB symptoms require further research (Garretto et al., 2018; Thomas et al., 2021). Additionally, human papillomavirus, adenovirus, and cytomegalovirus infections have been associated with LUTS (Paduch, 2007; Brubaker et al., 2021). Although urinary tract infections (UTIs) are mainly caused by bacteria such as *Escherichia coli* and viral infections are rare pathogens, viral infections of the urinary tract are linked to considerable morbidity and distress, including increased mortality in immunocompromised patients (Khonsari et al., 2021; Salabura et al., 2021). However, there is an important link between bacteria and viruses, such as increased antibiotic resistance in UTI patients, which leads to an increase in the global medical burden, and bacteriophages, as the most abundant group of human viruses, can be used as a feasible treatment for UTI (Behzadi et al., 2020; Ahmadi, 2021). It is important to note that, especially in female patients, misdiagnosis of UTI and genital tract infections (GTIs) may lead to imbalances in bacterial and viral populations and worsen disease (Behzadi et al., 2019).

Next-generation sequencing (NGS) of bacterial 16S rRNA is widely applied in clinical pathogen detection including urology (Derosa et al., 2020; Salabura et al., 2021). One such application is the unbiased next-generation metagenomic sequencing (mNGS), which overcomes the limitations of current diagnostic tests and allows untargeted, culture-independent pathogen detection directly from clinical samples (Neugent et al., 2020). At the same time, the development of bioinformatics for the analysis of sequencing data can help reduce possible biases in the results to obtain accurate and reliable analysis results (Behzadi and Ranjbar, 2018). Here, we seek to evaluate the role of urinary viruses in the onset of OAB using a mNGS approach, and attempt to reveal the potential relationship between viruses, bacteria, and OAB patients.

## Materials and methods

### Clinical procedures and sample acquisition

A total of 73 eligible individuals were identified from the outpatient clinic, including 55 OAB patients and 18 asymptomatic controls between January 2021 and August 2021. OAB patients fulfilling the following criteria were considered eligible for this study: (1) female, 18 years of age or older; (2) presented with classic OAB symptoms defined by the ICS; and (3) OAB Symptom Score (OABSS) total score ≥ 3 points and question 3 (urgency) score ≥ 2 points. The exclusion criteria include current urinary tract infection, with indwelling catheter, with antibiotic exposure in the past 30 days, chemical cystitis, cyclophosphamide cystitis, genitourinary cancer, urinary stones, neurogenic bladder, pelvic organ prolapse, pelvic radiation and pregnancy. The inclusion/exclusion criteria were consistent for the control subjects, except for the absence of OAB symptoms. The degree of OAB was appraised with both OABSS (mild ≤ 5, 6 < moderate < 11, severe ≥ 12) and 8-item OAB questionnaire (OAB-V8) during the recruitment. OAB patients were separated into the OAB virus-infected (OAB-VI) group and OAB virus-uninfected (OAB-VU) group based on the mNGS results. Similarly, the control group was divided into the control virus-infected group (CON-VI) and control virus-uninfected group (CON-VU).

As previously described, urine specimens were collected by transurethral catheterization to avoid contamination from distal urethra (Wolfe et al., 2012). In brief, with the patient in a lithotomy position, the perineum is thoroughly disinfected, followed by introducing a 16Fr catheter into the bladder to collect urine (Karstens et al., 2016; Yoo et al., 2021). Finally, 50 ml of urine was collected from each participant in this study, of which 20 ml was tested using standard urine culture methods to exclude UTI. The remaining 30 ml urine sample was temporarily stored in a 4°C refrigerator for 1 h and transferred to a −80°C freezer with DNA/RNA Shield (Zymo Research Corporation, Irvine, CA, United States) added until further processing. The study protocol was approved by the Institutional Review Board of Nanfang hospital (reference number: NFEC-2020-123).

### DNA extraction and metagenomic next generation sequencing

The DNA extraction was extracted using the TIANamp Micro DNA Kit (DP316, TIANGEN BIOTECH) sonicated to a size of 200–300 bps fragments (Bioruptor Pico protocols). Then, DNA libraries were constructed through DNA-fragmentation, end-repair, adapter-ligation and PCR amplification.

Quality qualified libraries were pooled. DNA Nanoball was made and sequenced by BGISEQ-50 platform. High-quality sequencing data was generated by removing low-quality reads, followed by computational subtraction of human host sequences mapped to the human reference genome (hg19) using Burrows–Wheeler Alignment tool^1^ (Li and Durbin, 2009). Low-quality reads refer to the basic characteristics that do not meet the following indicators: (1) the number of Q30 bases is more than 80%; (2) joint contamination ratio should not exceed 1%; (3) the effective sequence length is not <50 bp; and (4) the effective alignment rate of the data should be greater than 70% (Gu et al., 2019). The remaining data by removal of low-complexity reads were classified by simultaneously aligning to pathogens metagenomics Database (PMDB, BGI-Shenzhen), consisting of bacteria, fungi, viruses and parasites. We used a threshold of 10% minimum coverage of the viral genome or a viral stringently mapped reads number >100 for diagnosis (Jerome et al., 2019). The raw data of mNGS have been submitted to the Short Read Archive (PRJNA814640).

### Clinical data and bioinformatics analysis

Clinical characteristics between or among OAB and control groups were compared by the *t*-test or Mann–Whitney *U*-test for continuous variables, and by the Chi-square test for categorical variables. The Spearman correlation matrix was calculated to examine the pairwise between any two of all variables. Binary logistic regression was preformed and a predictive nomogram was drawn. All *p* values were two tailed, and the statistical significance level was set as 0.05. All statistical analyses were performed by R software (Version 4.1.1) and SPSS software (Version 21).

Kraken2 was used to annotate and classify all valid sequences of each sample to analyze microbial composition and diversity information (Wood and Salzberg, 2014). After using Kraken2 to classify mNGS data, Bracken 2.0 (default parameters) was used to estimate the relative abundance of metagenomic samples at different classification levels. Chao1 index, Shannon index and Simpson index calculated by QIIME2 were used to evaluate Alpha diversity. Through linear discriminant analysis (LDA) effect size (LEfSe) analysis, the differences of bacteria characteristics at all classification levels were identified (Segata et al., 2011).

### Phylogenetic tree analysis

We searched all available JC Virus complete genomes from the NCBI database (http://www.ncbi.nlm.nih.gov/, last accessed September 14, 2021) and combined with previous research databases that identified JC Virus subtypes and population distribution (Shackelton et al., 2006; Garretto et al., 2018; Forni et al., 2020). Next, 672 available JC Virus complete genomes and 24 JC Virus genomes identified through mNGS were compared, using MUSCLE to align the sequences through Geneious (Version 10.1.2). Subsequently, the maximum likelihood multi-model inference method was used to assign each query sequence to a reference sequence with known genotypes, followed by using the IQ-TREE to obtain a phylogenetic tree and the online tool Interactive Tree Of Life^2^ for annotation (Nguyen et al., 2015).

## Results

### The characteristics and microbiota composition of subjects: OAB vs. control

In total, 55 OAB patients and 18 asymptomatic controls were recruited in the study. There was no significant difference in the demographic characteristics between the two groups, with the exception of UTI history (60% vs. 17%, *p* = 0.002), pelvic surgery history (43% vs. 0%, *p* = 0.000) and the habit of holding urine (36% vs. 6%, *p* = 0.015, Table 1).

**Table 1:** The comparisons of demographic characteristics and symptom scores between OAB patients and Asymptomatic controls.

Characteristic	Overactive bladder (*n* = 55)	Asymptomatic controls (*n* = 18)	*p* Value^e^
Age, years	43.21 ± 14.55	50.5 ± 13.34	0.064
BMI, kg/m^2^	21.66 ± 3.51	22.68 ± 2.20	0.215
History of pregnancy, *n* (%)	49 (89)	17 (94)	0.670
Menopausal, *n* (%)	18 (33)	9 (50)	0.260
Previous estrogen treatment, *n* (%)	3 (5)	0 (0)	1.000
Hypertension, *n* (%)	9 (16)	3 (17)	1.000
Diabetes, *n* (%)	4 (7)	1 (6)	1.000
UTI history^a^, *n* (%)	33 (60)	3 (17)	0.002
Pelvic surgery history^b^, *n* (%)	24 (43)	0 (0)	0.000
Habit of holding urine^c^, *n* (%)	20 (36)	1 (6)	0.015
Habit of vulva clean^d^, *n* (%)	29 (53)	11 (61)	0.490
OABSS, scores	5.0 (4.0, 7.0)	1.0 (0.0, 1.0)	<0.001
Q1. Daytime frequency, scores	1.0 (1.0, 2.0)	1.0 (0.0, 1.0)	<0.001
Q2. Nighttime frequency, scores	1.0 (1.0, 2.0)	0.0 (0.0, 0.0)	<0.001
Q3. Urgency, scores	2.0 (2.0, 4.0)	0.0 (0.0, 0.0)	<0.001
Q4. Urgency incontinence, scores	0.0 (0.0, 0.0)	0.0 (0.0, 0.0)	0.054
OAB-V8, scores	17.0 (10.0, 23.0)	1.0 (0.0, 1.0)	<0.001

BMI, body mass index.

a UTI history, at least one-time UTI during her lifetime.

b Pelvic surgery history, non-urologic pelvic surgery, including hysterectomy, dilatation and curettage, colorectal resection and pelvic fracture.

c Habit of holding urine, often wait until the last second before going to the bathroom to pee.

d Habit of vulva clean, use of non-irritating agent (water) or antiseptic solution for genital cleaning in daily life.

e *p* Values were calculated using Wilcoxon rank-sum test for continuous and Fisher’s exact test for categorical variables. The data are expressed as the means ± standard deviations or medians (interquartile ranges).

**Figure 1:**
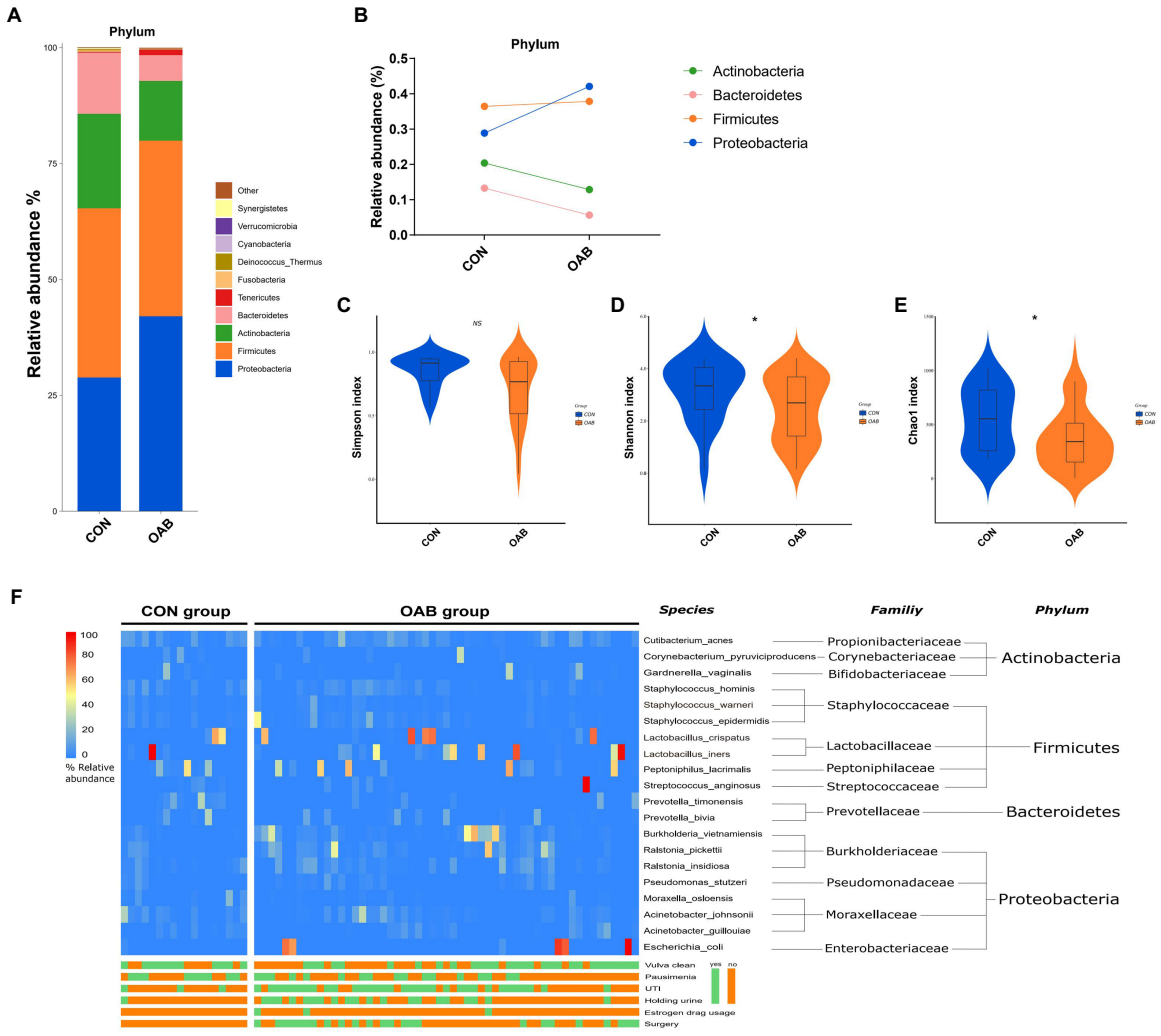
The urinary microbiome profile of participants. **(A)** The bar graph showing the relative abundance of urine microbiota at phylum level. **(B)** Composition Changes of Actinobacteria, Bacteroidetes, Firmicutes and Proteobacteria between the control and OAB groups. Alpha diversity analysis for the control and OAB urinary microbiomes including Simpson index **(C)**, Shannon index **(D)** and Chao1 index **(E)**. **(F)** Heatmap demonstrates the relative abundance of the microbiota at different levels of the control and OAB groups and clinical data.

**Figure 2:**
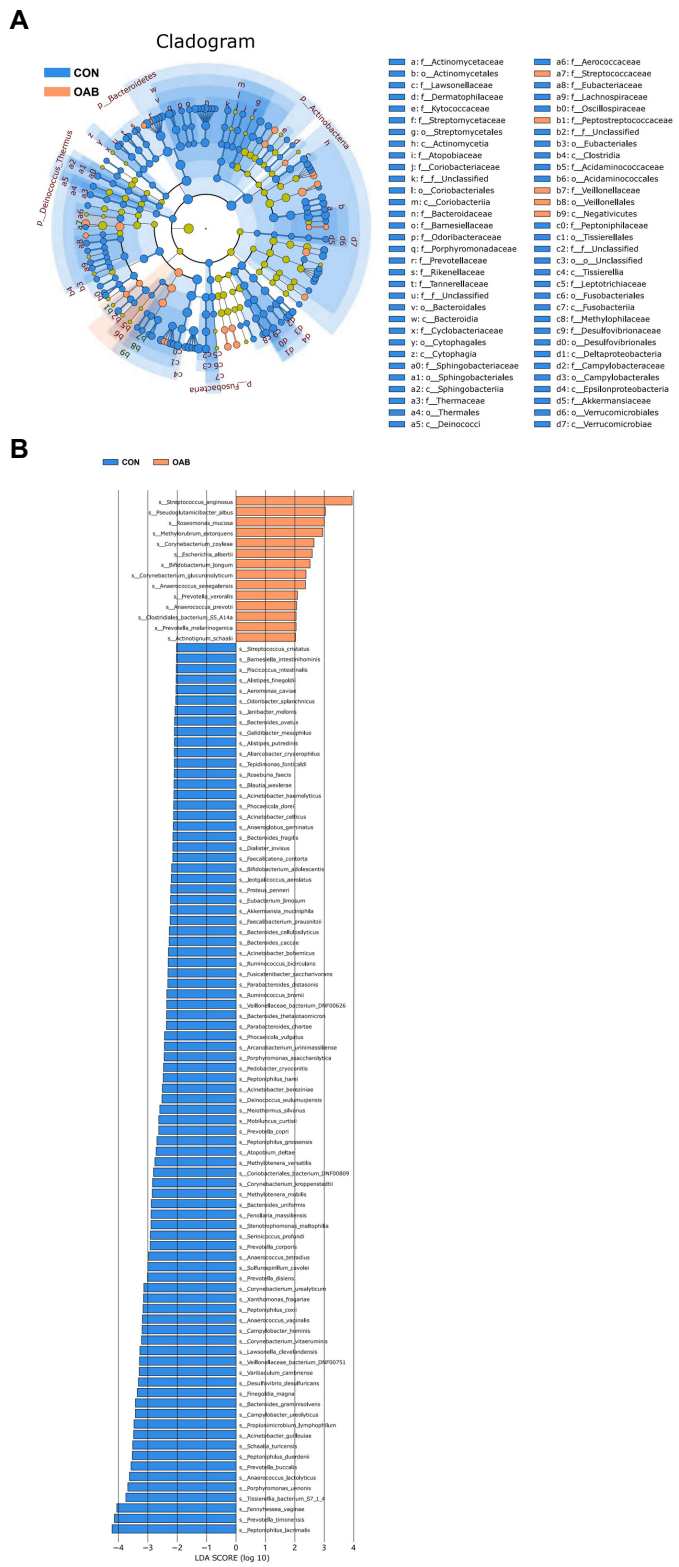
Microbial taxa associated with Overactive bladder. **(A)** Cladogram representation of the urinary microbial taxa associated with OAB (yellow red) and control (blue). **(B)** Association of specific microbiota taxa with OAB group and control group by linear discriminant analysis effect size (LEfSe). Yellow red indicates taxa enriched in OAB group and blue indicates taxa enriched in control group. Only species meeting a linear discriminant analysis score threshold >2 are shown.

A total of 5,883,488 bacterial reads were obtained. At the phylum level, Proteobacteria, Firmicutes, Actinobacteria and Bacteroidetes were the most common types in both groups, with Bacteroidetes decreased significantly in patients with OAB (*p* = 0.0005, Figures 1A,B). Although the Simpson index (*p* = 0.056, Figure 1C) was similar, the Shannon index and chao1 index were significantly reduced in the OAB group at the species level (*p* = 0.043, Figure 1D; *p* = 0.022, Figure 1E). The clinical information corresponding to the urine sample and microbial composition and relative abundance are shown in Figure 1F, which is intuitive to show that *Lactobacillus inner*, *Burkholderia vietnamiensis and Escherichia coli* are enriched in the OAB group. To identify specific OAB related bacteria, the LEfSe algorithm was used, indicating significantly elevated compositional abundance of 14 species in OAB patients and other 85 species in control subjects (Figure 2).

### The characteristics and microbiota composition of OAB patients with or without urinary virus

**Figure 3:**
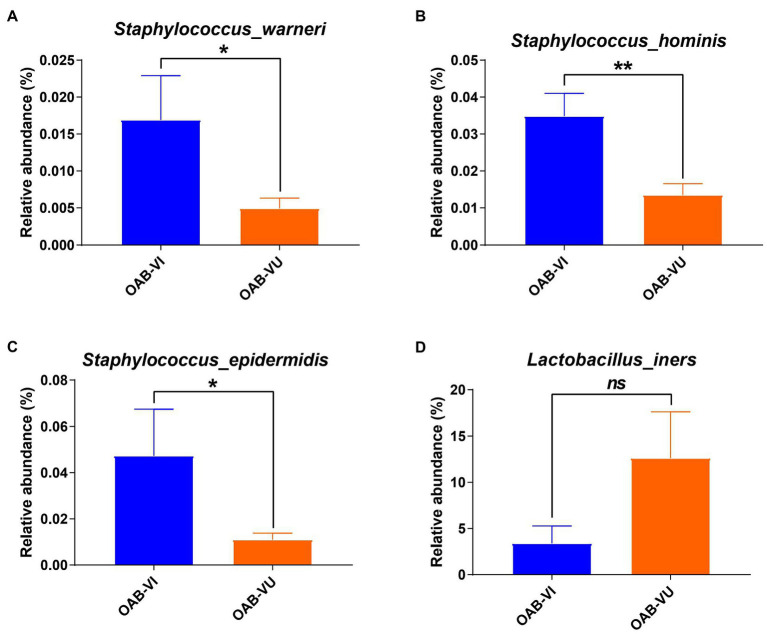
Bacterial average relative abundance in OAB-VI and OAB-VU. Relative abundance of **(A)** *Staphylococcus warneri*, **(B)** *Staphylococcus hominis*, **(C)** *Staphylococcus epidermidis* and **(D)** Lactobacillus inner. *p≤0.05, **p≤0.01.

Twenty-six OAB patients (47.3%) identified with positive urinary virus were classified as OAB-VI group and the remaining 29 patients (52.7%) without detectable virus were allocated to the OAB-VU group (Supplementary Table 1). Notably, patients in the OAB-VI group demonstrated much worse OAB symptoms than that in the OAB-VU group as evidenced by both the OABSS and OAB-V8 questionnaires (Table 2). Plus, there were significantly more moderate-to-severe cases in the OAB-VI group, while mild OAB symptoms were predominant if without virus detection (Supplementary Figure S1). Crucially, at species level, the relative abundance of *Staphylococcus warneri* (*p* = 0.0092), *Staphylococcus hominis* (*p* = 0.0017) and *Staphylococcus epidermidis* (*p* = 0.0041) was significantly increased in the OAB-VI group (Figures 3A–C; Supplementary Figure S2). In contrast, there was no significant difference in *Lactobacillus inner* richness in OAB-VI compared with OAB-VU, but there was a downward trend (Figure 3D).

**Table 2:** The comparison of clinical features of OAB patients with or without viral infection.

Characteristic	OAB-VI (*n* = 26, 47%)	OAB-VU (*n* = 29, 53%)	*p* Value^e^
Age, years	47.5 ± 15.04	39.5 ± 12.26	0.039
BMI, kg/m^2^	22.68 ± 3.78	20.77 ± 3.07	0.041
Ever pregnant, *n* (%)	24 (92)	25 (83)	0.431
Menopausal, *n* (%)	12 (46)	6 (20)	0.037
Estrogen treatment, *n* (%)	2 (8)	1 (3)	0.592
Hypertension, *n* (%)	5 (19)	4 (13)	0.719
Diabetes, *n* (%)	2 (8)	2 (7)	1.000
UTI history^a^, *n* (%)	21 (81)	12 (40)	0.002
Pelvic surgery history^b^, *n* (%)	16 (62)	8 (27)	0.009
Habit of holding urine^c^, *n* (%)	14 (54)	6 (20)	0.008
Habit of vulva clean^d^, *n* (%)	7 (27)	22 (73)	0.001
OABSS, scores	7.0 (6.0, 9.0)	4.5 (4.0, 5.3)	<0.001
Q1. Daytime frequency, scores	1.5 (1.0, 2.0)	1.0 (1.0, 1.0)	0.003
Q2. Nighttime frequency, scores	1.81 ± 1.02	1.37 ± 0.93	0.096
Q3. Urgency, scores	4.0 (3.0, 5.0)	2.0 (2.0, 2.6)	<0.001
Q4. Urgency incontinence, scores	0.0 (0.0, 2.0)	0.0 (0.0,0.0)	0.060
OAB-V8, scores	21.58 ± 7.44	14.20 ± 6.97	<0.001

BMI, body mass index.

a UTI history, at least one-time UTI during her lifetime.

b Pelvic surgery history, non-urologic pelvic surgery, including hysterectomy, dilatation and curettage, colorectal resection and pelvic fracture.

c Habit of holding urine, often wait until the last second before going to the bathroom to pee.

d Habit of vulva clean, use of non-irritating agent (water) or antiseptic solution for genital cleaning in daily life.

e *p* Values were calculated using Wilcoxon rank-sum test for continuous and Fisher’s exact test for categorical variables. The data are expressed as the means ± standard deviations or medians (interquartile ranges).

**Figure 4:**
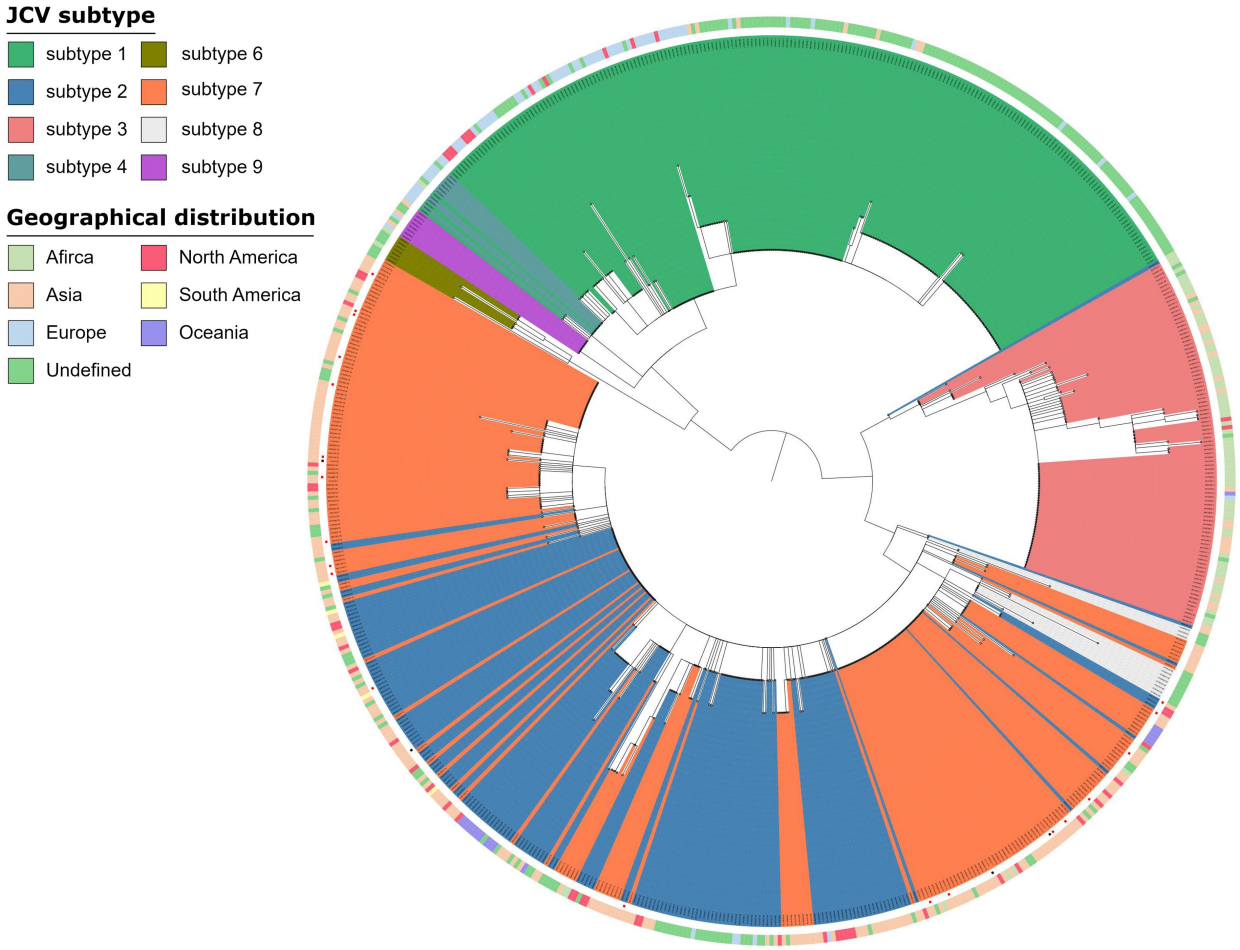
Phylogenetic analysis JC virus. Phylogenetic tree of 672 JC Virus complete genomes inferred using a maximum likelihood approach, including strains in this study (a red square at the end of a branch indicates). The genomes from OAB-VI (red squares) and control (black squares) subjects were inserted into the circular dendrite map. The tree branch color represents different types and peripheral color bands represent geographic distribution. Samples analyzed in the study are labeled with the sample name and JC Virus subtype.

Virus genomes were detected in 32 of 73 samples, of which 26 samples were from the OAB group and 6 samples were from the control group (Supplementary Table 1). JC virus was the most common of all viral genomes (*n* = 25), BK virus, EB virus and human beta-herpes virus 6A were also detected (Supplementary Table 1). However, the JC virus genome detected in urine sample of OAB-VI25 was not included because the coverage rate was too low to be classified (Supplementary Table 1). The main subtype of JC Virus strains isolated in the samples were mainly subtype 7B (OAB, *n* = 8; control, *n* = 1) and subtype 7A (OAB, *n* = 7; control, *n* = 2) followed by subtype 7C (OAB, *n* = 2; control, *n* = 0), subtype 2D (OAB, *n* = 2; control, *n* = 0) and subtype 2A (OAB, *n* = 1; control, *n* = 1). Based on the geographic distribution of these virus subtypes, we found that they were mainly from the Asian region (*n* = 19, Figure 4).

Finally, by comparing alpha diversity of the CON-VI, CON-VU, OAB-VI and OAB-VU groups, we only found that the Chao1 index of the OAB-VI group was significantly lower than that of the CON-VU group (Supplementary Figures S3A–C).

### Correlation analysis between OAB symptoms and risk factors for viral infection in OAB patients

**Figure 5:**
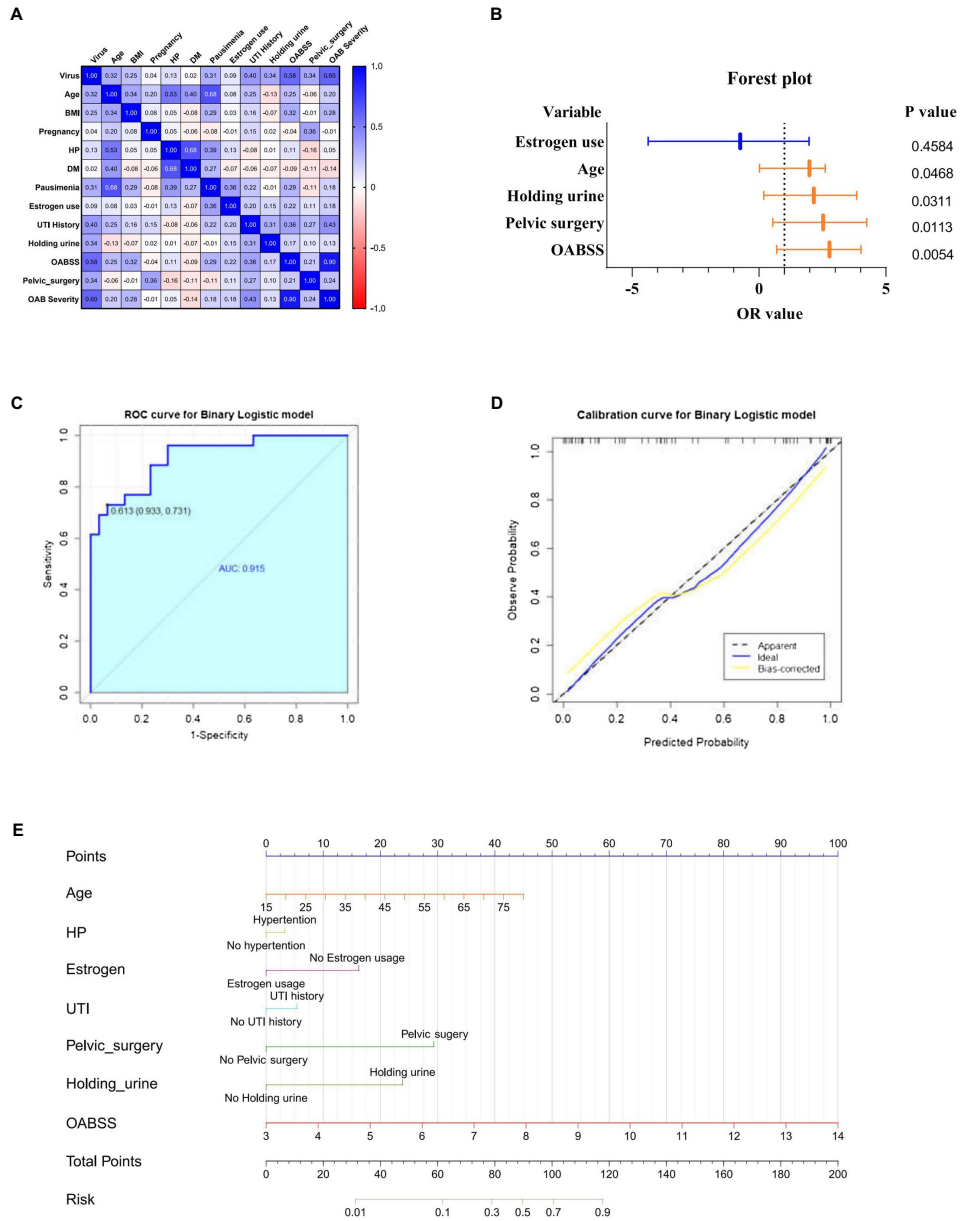
Correlation analyze with OAB symptoms and risk factors for viral infection. **(A)** Heatmap for pearson correlation coefficient (PCCs) matrix for virus infection, age, BMI, pregnancy, HP, DM, pausimenia, estrogen use, UTI history, habit of holding urine history, OABSS, age group, BMI group, Pelvic surgery, OAB severity (*n* = 55). **(B)** Forest plot for screened risk factor and protective factor of viral infection (*p* < 0.05). **(C)** Receiver operating characteristic curve (ROC curve): area under the ROC curve is 0.915 (*p* < 0.001). **(D)** Nomogram for the prediction of viral infection. Instruction for utilization: locate the patient’s age on the corresponding axis. Draw a line straight down to the point axis to determine how many points toward the probability of viral infection. Repeat the process for each additional predictor and add up all the points. Locate the final sum on the total points axis. Draw a line straight up to find the patient’s risk of viral infection so as to provide guidance of mNGS application. **(E)** Calibration curve for the nomogram to predict probability of viral infection.

Spearman correlation analysis was used to identify intra-variable linear correlation between variables, results indicated existence of high correlations between viral infection and OABSS (*r* = 0.58), age and pausimenia (*r* = 0.68), hypertension and age (*r* = 0.53) respectively (Figure 5A). In addition, viral infection is associated with over 10 times more likely to have a worse OAB symptom (Table 3).

**Table 3:** OR value calculation of risk factors for OAB symptom severity.

Parameter	OR value	95% Confidence interval
Virus infection	10.40	2.99, 36.25
Ever pregnant	1.23	0.23, 5.59
Estrogen treatment	2.52	0.22, 29.58
Hypertension	1.24	0.28, 5.55
Diabetes	0.38	0.04, 3.85
Urinary tract infection	5.23	1.55,1 7.67
Pelvic surgery	1.87	0.64, 5.51
Habit of holding urine	2.54	0.82, 7.84

OABSS, mild ≤ 5, 6 < moderate < 11, severe ≥ 12; Moderate and severe are jointed in calculation of OR value to minimize the effect of unequal distance between hierarchical variable and small sample size of severe (*n* = 3). The data are expressed as OR value [95%CI (lower limit of ratio, higher limit of ratio)].

Estimates from binary logistic regression model indicated risk factors for virus infection in OAB patients including age (OR = 1.99; 95%CI: 0.02, 2.61), holding urine habit (OR = 2.16; 95%CI: 0.18, 3.85) and pelvic surgery (OR = 2.53; 95%CI: 0.54, 4.27). Protective factors include estrogen use history (OR = −0.74; 95% CI: −4.37, 1.97) (Table 4; Figure 5B). Five clinical variables with values greater than 1 are: virus infection (VIP = 1.94), UTI history (VIP = 1.21), BMI (VIP = 1.13), pausimenia (VIP = 1.02, Supplementary Figure S4). The area under ROC curve is 0.915 (*p* < 0.0001) for our binary logistic regression predictive model (Figures 5C,D). Then, nomogram predictive model based on visualized binary logistic regression result was built for virus infection prediction, including patient history of hypertension, estrogen usage, UTI history, pelvic surgery history, the habit of holding urine as independent predictive factors for virus infection (Figure 5E).

**Table 4:** Risk factors for virus infection identified by binary logistic regression.

Risk factors	Odds ratio	95% Confidence interval	*p* Value
Age	1.99	0.0187, 2.6088	0.0468
Estrogen use	−0.74	−4.3713, 1.9717	0.4584
OABSS	2.78	0.698, 4.0217	0.0264
Habit of holding urine	2.16	0.1833, 3.8479	0.0311
Pelvic surgery	2.53	0.5409, 4.2464	0.0113

## Discussion

The correlations between alteration of urine microbiome and occurrence of OAB have not been revealed until recent years (Wolfe et al., 2012; Pearce et al., 2014; Wu et al., 2017; Brubaker et al., 2021). We have previously demonstrated that at the genus level, the abundance of *Aerococcus* and *Staphylococcus* increased, while *Prevotella* and *Lactobacillus* decreased in OAB patients (Wu et al., 2017). By comparing the urinary microbiome of women with UUI and asymptomatic controls, Pearce et al. found that the microbiome from UUI showed a decreased in *Lactobacillus* and an increased in *Grdnerella* abundance from the UUI cohort by EQUC (Pearce et al., 2014). Additionally, Karstens et al. (2016) determined that an increased severity of UUI symptoms was associated with decreased microbial diversity in female UUI patients. However, most studies on urinary microbiome were measured by 16S rRNA gene sequencing, and investigations were limited to the bacteria. In the present study, we took the lead in unveiling the effects of urinary viral infection on the presence of OAB, and providing evidence on the connections between modified microbial features and degree of OAB symptoms.

Consistent with our previous report, the analysis of Chao1 and Shannon index indicated that the OAB group had lower diversity than the control group, which was consistent from both 16S rRNA gene sequencing and mNGS results (Wu et al., 2017). Since most studies on the urinary microbiome used 16S rRNA amplicon sequencing and focused on the genus level analysis, few studies have similar results at the species level. Recently, Wolfe et al. have provided new insights into taxonomic schemes for species-level bacterial annotations in 16S amplicon data (Hoffman et al., 2021). In the results obtained by mNGS, at species level, the OAB-VI group demonstrated significantly decreased *Lactobacillus iners*, but increased coagulase-negative Staphylococci (CoNS), including *Staphylococcus epidermidis*, *Staphylococcus hominis* and *Staphylococcus warner*. Previous studies of *Staphylococcus warneri*, *Staphylococcus epidermidis* and *Staphylococcus hominis* are mainly elaborated in human skin microbiome (Byrd et al., 2018). Staphylococcus epidermidis may act both as an opportunistic pathogen and a probiotics, which prevents bacterial biofilm formation *via* expression of a serine type protease, termed Esp (Otto, 2009; Sugimoto et al., 2013). On the contrary, known as the most sensitive microbiome marker in urinary tract diseases, a decreased *Lactobacillus* in the urine was associated with the presence of OAB/UUI (Wu et al., 2017; Peyronnet et al., 2019). In a study, whole-genome phylogenetic analysis of bacterial strains isolated from the vagina and bladder of the same female identified highly similar *Lactobacillus iners* and *Lactobacillus crispatus*, both of these *Lactobacillus* have been linked to health in the vagina, as well as the bladder in asymptomatic women (Thomas-White, 2018). These conclusions may indicate its potential role in prevention and treatment of OAB as a probiotics.

Viral genomes, especially JC Virus and BK Virus, were detected predominantly more frequent in patients with OAB. Similarly, Garretto et al. (2018) found that urine samples from OAB patients contain complete genomes of JC Virus while no viral genes were detected in the control group. In reality, JC Virus may infect healthy persons latently, resulting in asymptomatic replication and sporadic detection of the virus in the urine in up to 30% of the general population (White and Khalili, 2011). Previous studies have determined the subtype of JC Virus and global distribution (Forni et al., 2020). The main subtype of JC Virus strains detected in urine of OAB patients were subtype 7B (*n* = 8) and subtype 7A (*n* = 7) and these two subtypes of JC Viruses are mainly prevalent in Asia, which was obviously inconsistent with the subtype measured by Wolfe et al., since their samples were mainly from Caucasians and African Americans (Garretto et al., 2018). Interactions between virus and bacteria are widely reported in gastrointestinal tract, mostly focusing on the detrimental effects of bacteriophage on bacteria and end up with reduce in specific bacteria (Santiago-Rodriguez and Hollister, 2019). The bacterial microbiota is required for many enteric viruses during the infection of eukaryotic cells (Monedero et al., 2018). The effects of bacteria on eucaryotic viruses include direct binding with virions to enhance stability and indirect effects led by immune activation through the attachment of host cells (Rowe et al., 2019). Nevertheless, the evidence on whether the virus interacts directly or indirectly with urinary tract bacteria remains to be discovered.

In a cross-sectional study, women with unhealthy habit of holding urine suffer from more frequent LUTS, and this feature was also found in our clinical practice (Reynolds et al., 2020). Here, we found that history of pelvic surgery, holding urine habit and UTI history are risk factors for viral infection, which may make the symptoms of OAB worse. However, GTIs was not taken into account in this study. The association between history of GTIs and OAB and viral infection in women deserves further exploration (Behzadi et al., 2019, 2020). Estrogen usage history whose protective impact of vaginal estrogen therapy has been reported on postmenopausal OAB patients associated with increase of Lactobacillus is found to be a protective factor of viral infection (Thomas-White et al., 2020). Patients in the virus infected group present more severe symptoms and received higher OABSS and OAB-V8 score, which could be explained by the bladder epithelium damage caused by JC or BK viral replication (Li et al., 2013). Given the multifactorial nature of OAB, further research and clinical observations to identify the triple relationship among OAB, virus, bacteria as well as the importance of viral detection and targeted treatment in OAB patients would greatly benefit clinical practice.

## Conclusion

In conclusion, we focused at the bacterial and viral composition of urine from OAB and healthy individuals, and found that more severe symptoms of OAB patients seems to be related to viral infection. This is the first published research on the function of human urine bacteria and viruses in OAB female patients that we are aware of. It is our hope that this work lays the foundation for future research on female urine microbiota and OAB disease.

## Data availability statement

The datasets presented in this study can be found in online repositories. The names of the repository/repositories and accession number(s) can be found in the article/Supplementary material.

## Author contributions

PW, JZ, and QSo: study concept and design. PW, HZ, YG, BW, YQ, and ZZ: investigation. PW, HZ, and BW: supervision. HZ, QSu, LL, and YW: analysis and interpretation of data. PW, QSo, QSu, and LL: writing – original draft. PW, JZ, QSo, and YW: writing – review and editing. PW: funding acquisition. All authors contributed to the article and approvedthe submitted version.

## Funding

This current study was supported by funding from the National Natural Science Foundation of China (grant nos. 81870522 and 82173304) and the Natural Science Foundation of Guangdong Province (grant nos. 2018A030313148 and 2021A1515012262).

## Conflict of interest

The authors declare that the research was conducted in the absence of any commercial or financial relationships that could be construed as a potential conflict of interest.

## Publisher’s note

All claims expressed in this article are solely those of the authors and do not necessarily represent those of their affiliated organizations, or those of the publisher, the editors and the reviewers. Any product that may be evaluated in this article, or claim that may be made by its manufacturer, is not guaranteed or endorsed by the publisher.

## Footnotes

^1^ http://bio-bwa.sourceforge.net/

^2^ https://itol.embl.de/
