# Supplementary figures and images for: The detection of urinary viruses is associated with aggravated symptoms and altered bacteriome in female with overactive bladder
Source: Front Microbiol. 2022 Sep 23;13:984234. doi: 10.3389/fmicb.2022.984234 (PMC9537457; doi:10.3389/fmicb.2022.984234)

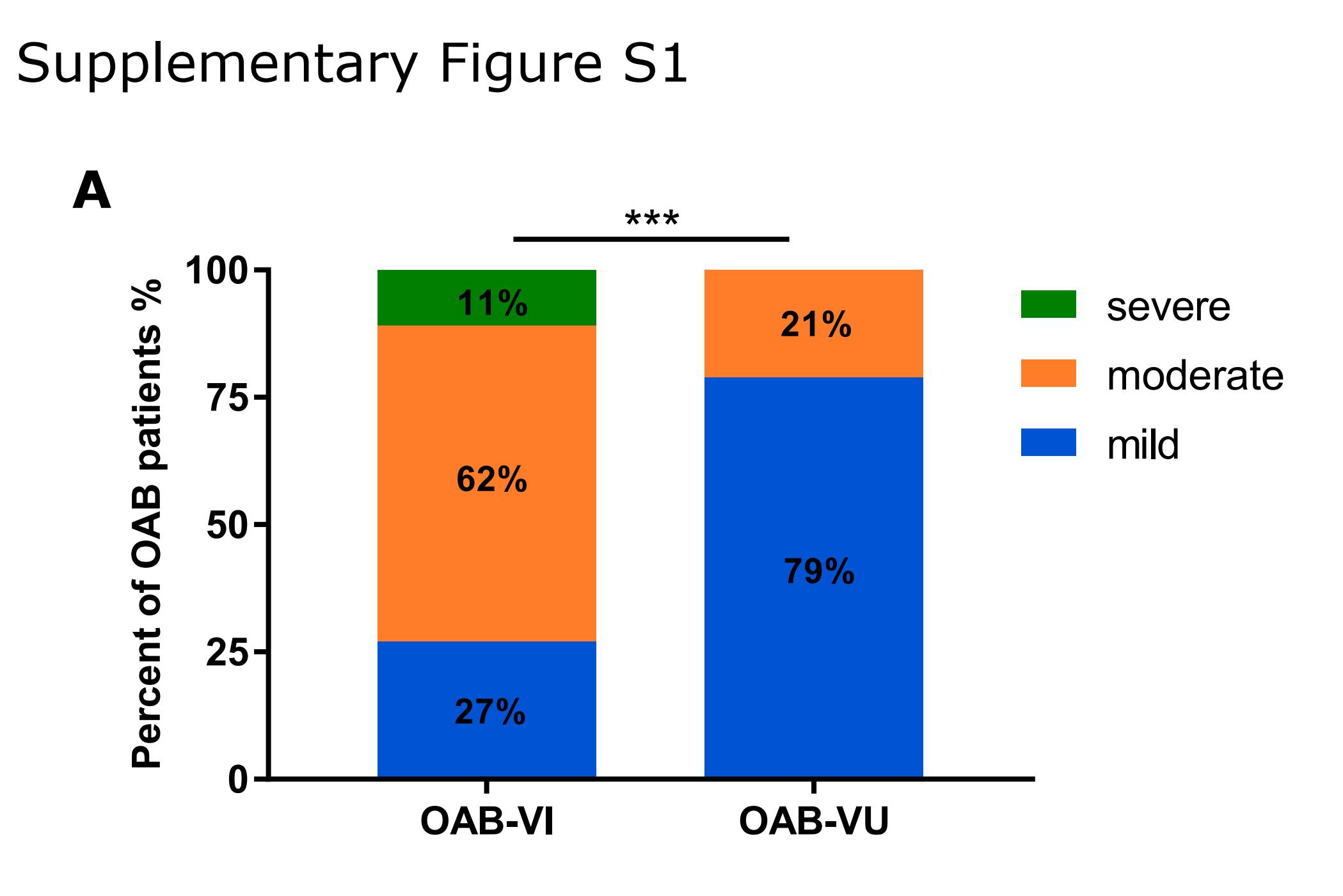

Supplement: Supplementary file 1 [file Image_1.JPEG]

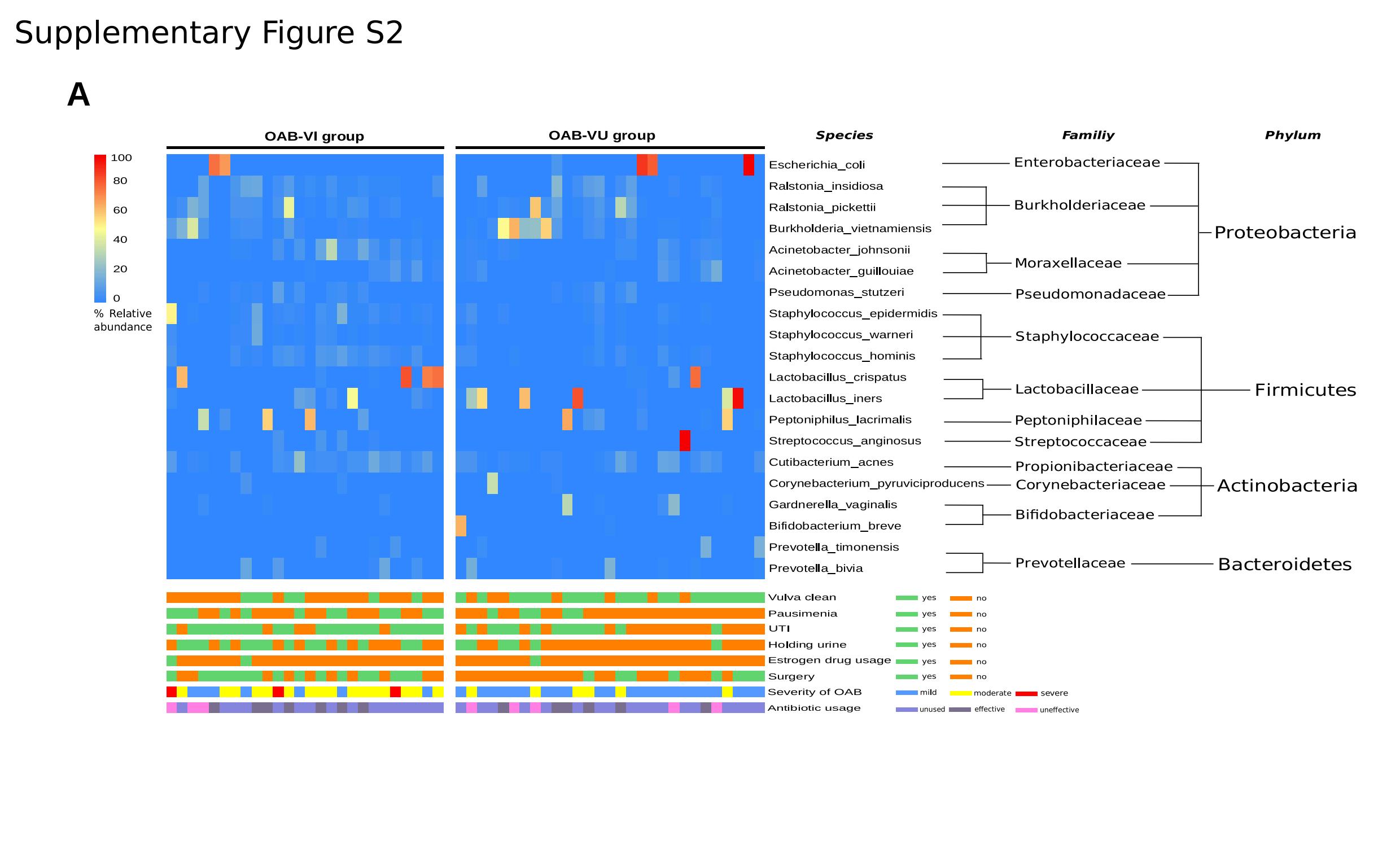

Supplement: Supplementary file 2 [file Image_2.JPEG]

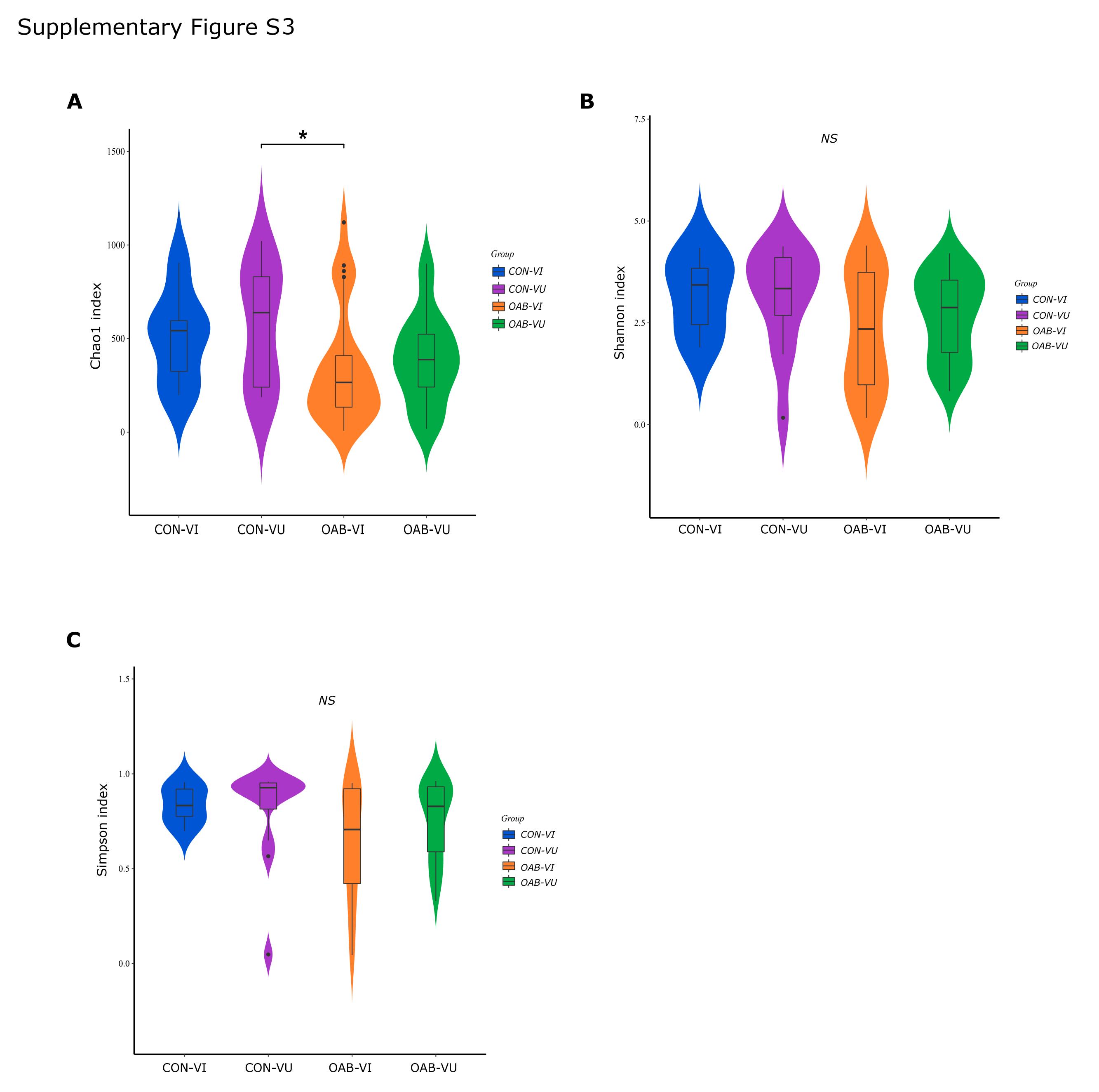

Supplement: Supplementary file 3 [file Image_3.JPEG]

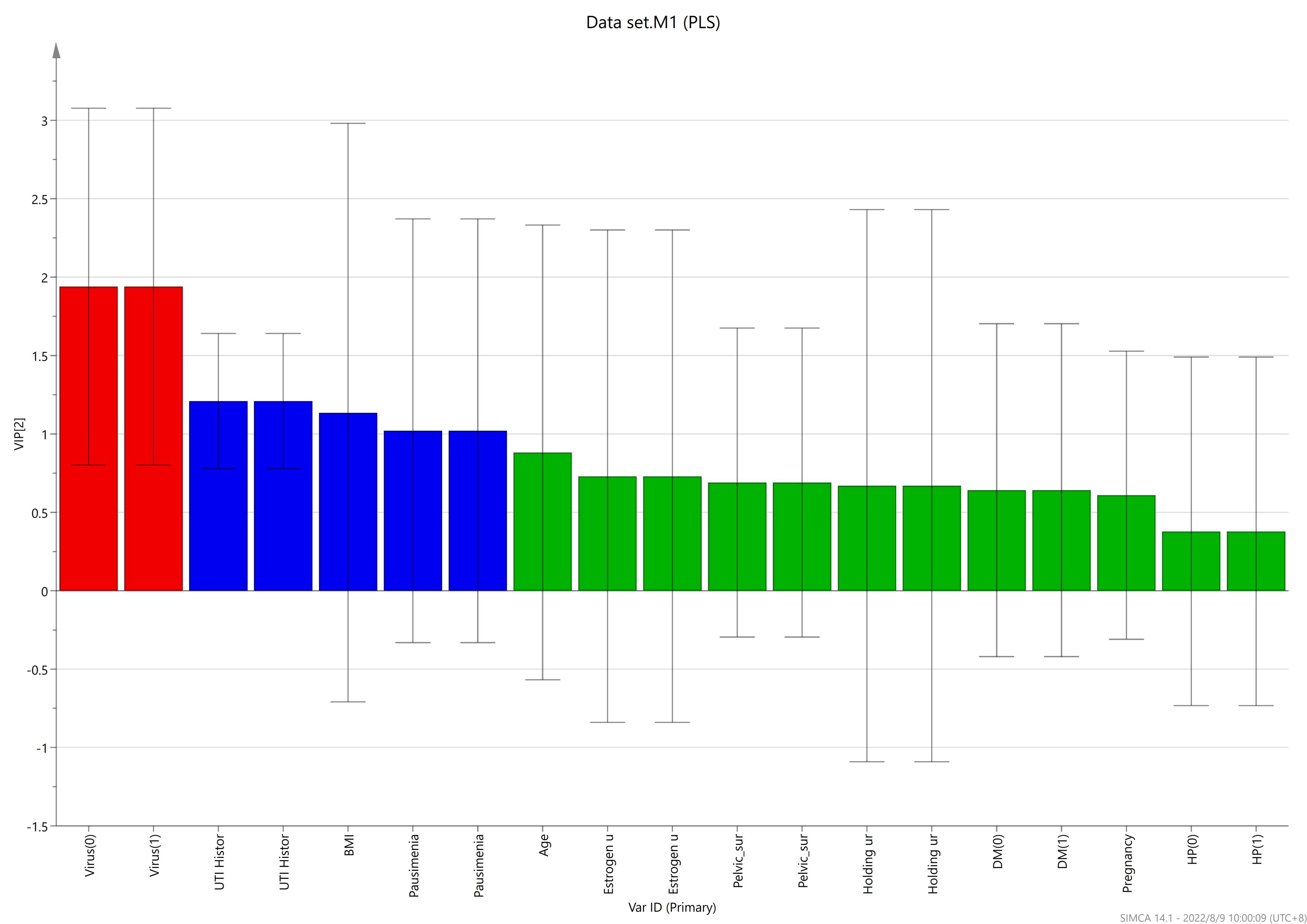

Supplement: Supplementary file 4 [file Image_4.JPEG]
