# Supplementary material for: The detection of urinary viruses is associated with aggravated symptoms and altered bacteriome in female with overactive bladder
Source: Front Microbiol. 2022 Sep 23;13:984234. doi: 10.3389/fmicb.2022.984234 (PMC9537457; doi:10.3389/fmicb.2022.984234)
Supplement: Supplementary file 5 [file Table_1.docx]

**Supplementary table 1: Virus parameters in 32 urine samples from mNGS.**

| Sample | species of virus | Coverage Rate (%) | Depth | SMRN | Relative Abundance (%) |
| --- | --- | --- | --- | --- | --- |
| OAB-VI1 | JC virus | 86.75 | 3.79 | 398 | 99.29 |
| OAB-VI2 | JC virus | 89.99 | 5.02 | 569 | 99.95 |
| OAB-VI3 | BK virus | 63.16 | 1.79 | 124 | 100 |
| OAB-VI4 | JC virus | 88.10 | 3.59 | 373 | 98.61 |
| OAB-VI5 | JC virus | 71.21 | 2.34 | 200 | 100 |
| OAB-VI6 | JC virus | 98.84 | 77.32 | 14594 | 99.84 |
| OAB-VI7 | JC virus | 94.66 | 43.79 | 5651 | 100 |
| OAB-VI8 | human beta-herpesvirus 6A | 7.9 | 1.03 | 178 | 100 |
| OAB-VI9 | BK virus | 35.41 | 1.23 | 50 | 100 |
| OAB-VI10 | EB virus | 96.22 | 8.56 | 6321 | 100 |
| OAB-VI11 | BK virus | 96.89 | 5.16 | 600 | 100 |
| OAB-VI12 | JC virus | 96.93 | 41.17 | 6104 | 99.98 |
| OAB-VI13 | JC virus | 98.82 | 70.31 | 19042 | 99.61 |
| OAB-VI14 | JC virus | 96.80 | 28.80 | 4049 | 99.78 |
| OAB-VI15 | JC virus | 95.21 | 39.41 | 3938 | 99.13 |
| OAB-VI16 | JC virus | 97.71 | 48.39 | 7141 | 99.87 |
| OAB-VI17 | JC virus | 96.47 | 29.67 | 4281 | 99.86 |
| OAB-VI18 | JC virus | 99.87 | 73.12 | 29660 | 97.74 |
| OAB-VI19 | JC virus | 94.53 | 13.01 | 1725 | 100 |
| OAB-VI20 | JC virus | 95.98 | 24.57 | 3447 | 100 |
| OAB-VI21 | JC virus | 82.00 | 2.98 | 345 | 99.47 |
| OAB-VI22 | JC virus | 99.03 | 68.80 | 16886 | 99.48 |
| OAB-VI23 | JC virus | 97.03 | 51.04 | 7735 | 100 |
| OAB-VI24 | JC virus | 94.81 | 16.88 | 2313 | 100 |
| OAB-VI25 | JC virus | 16.82 | 1.07 | 30 | 90.98 |
| OAB-VI26 | JC virus | 96.03 | 22.29 | 3102 | 94.3 |
| Control-1 | JC virus | 83.68 | 3.47 | 411 | 99.19 |
| Control-3 | JC virus | 99.18 | 71.25 | 27199 | 94.99 |
| Control-13 | BK virus | 99.59 | 67.41 | 9840 | 99.41 |
| Control-15 | JC virus | 98.02 | 59.24 | 10140 | 99.04 |
| Control-16 | BK virus | 90.54 | 6.36 | 856 | 100 |
| Control-17 | JC virus | 93.61 | 10.38 | 1368 | 100 |
| SMRN: Stringently Mapped Reads Number | | | | | |
